# Variable resource allocation pattern, biased sex-ratio, and extent of sexual dimorphism in subdioecious *Hippophae rhamnoides*

**DOI:** 10.1371/journal.pone.0302211

**Published:** 2024-04-18

**Authors:** Manisha Jhajhariya, Yash Mangla, Atika Chandra, Shailendra Goel, Rajesh Tandon

**Affiliations:** 1 Department of Botany, University of Delhi, New Delhi, India; 2 Department of Botany, Kirori Mal College, University of Delhi, New Delhi, Delhi, India; 3 Department of Botany, Maitreyi College, University of Delhi, New Delhi, Delhi, India; Sant Baba Bhag Singh University, INDIA

## Abstract

Evolutionary maintenance of dioecy is a complex phenomenon and varies by species and underlying pathways. Also, different sexes may exhibit variable resource allocation (RA) patterns among the vegetative and reproductive functions. Such differences are reflected in the extent of sexual dimorphism. Though rarely pursued, investigation on plant species harbouring intermediate sexual phenotypes may reveal useful information on the strategy pertaining to sex-ratios and evolutionary pathways. We studied *H*. *rhamnoides* ssp. *turkestanica*, a subdioecious species with polygamomonoecious (PGM) plants, in western Himalaya. The species naturally inhabits a wide range of habitats ranging from river deltas to hill slopes. These attributes of the species are conducive to test the influence of abiotic factors on sexual dimorphism, and RA strategy among different sexes. The study demonstrates sexual dimorphism in vegetative and reproductive traits. The sexual dimorphism index, aligned the traits like height, number of branches, flower production, and dry-weight of flowers with males while others including fresh-weight of leaves, number of thorns, fruit production were significantly associated with females. The difference in RA pattern is more pronounced in reproductive traits of the male and female plants, while in the PGM plants the traits overlap. In general, habitat conditions did not influence either the extent of sexual dimorphism or RA pattern. However, it seems to influence secondary sex-ratio as females show their significant association with soil moisture. Our findings on sexual dimorphism and RA pattern supports attributes of wind-pollination in the species. The observed extent of sexual dimorphism in the species reiterates limited genomic differences among the sexes and the ongoing evolution of dioecy via monoecy in the species. The dynamics of RA in the species appears to be independent of resource availability in the habitats as the species grows in a resource-limited and extreme environment.

## Introduction

Evolution of unisexuality and dioecy among flowering plants is a complex phenomenon, as the variables contributing to its manifestation may differ from those that facilitate its maintenance. Theoretical models and empirical studies have held the importance of hermaphroditic origin for the evolution of dioecy, which leads to separation of sex expression at the flower or individual level [1, 2]. This separation is accompanied by organization of sex-specific features termed primary sex characters [3]. Empirical studies have also established that there are two major transitory routes of the evolution of dioecy namely, gynodioecy and monoecy. The transition may often leads to appearance of a range of intermediate sex phenotypes like subdioecy, cryptic dioecy etc. [3]. Such intermediate sex phenotypes have proved valuable in characterizing the pathways to dioecy among plants, and provide an opportunity to test the benefits and cost of unisexuality vs. hermaphroditism and their functional role [4].

The initial maintenance of unisexuality may rely on gender determination mechanism, and pollination mode [5–7]. Once the transitory route to dioecy is established, its maintenance and regulation may also be governed by a variety of biotic and abiotic factors [8, 9]. The mechanistic of such a dynamism can be context-dependent and is largely seen to be governed by interaction between species (*sensu stricto* species-specific characters) and the environment [8, 10, 11]. Some studies have shown that soil nutrient availability, moisture, temperature, and light conditions can significantly modulate the availability of resources and eventually influence spatial segregation of sexes [12, 13]. It is also opined that spatial heterogeneity in microhabitats of sexes is likely to be linked with trade-off in their resource allocation between the vegetative and reproductive functions [14, 15]. The male and female plants may manage different strategies for both the acquisition and partitioning of resources [16–18]. Further, the differential resource allocation pattern among sexes leads to variability in their life-history traits as well like, vegetative growth, clone size, phenology, floral traits, and flower production. Such a variability is regarded as sexual dimorphism and leads to emergence of secondary-sex characters and sexual-size dimorphism [14, 18, 19]. These suites of characters are believed to aid in better maintenance of dioecy through effective gene dispersal and its fixation [7, 20].

Sex-based differences, both in traits as well as in resource allocation (RA) pattern, are important factors which determine their respective performance in varying habitats and may provide a chance to study sex biases [4, 10]. For example, in perennial and gynodioecious *Leucopogon melaleucoides*, females exhibit two-fold fruit-set and greater allocation of resources at the time of fruiting than that in the hermaphrodites. The study highlighted that sexual dimorphism in investigated traits and RA pattern is less pronounced in gynodioecious species with an implication on spread and establishment of females in the population [21]. Likewise, in *Sagittaria latifolia*, which shows a continuous variation in sex phenotypes, the frequency of hermaphrodite plants is shown to increase in comparison to female plants owing to relatively low-cost of reproduction [4]. Thus, the relative proportion of sexes in a population is an indication of the evolutionary, ecological, and genetic stability for diclinous species [22, 23].

Studies have also indicated that the sex-ratios in natural populations are an interplay between demographic factors and underlying sex determination mechanism [24, 25]. Usually, in dioecious taxa with sex-chromosomes based mechanism (especially XY), gender segregation during gamete formation is expected to be 1:1 (male:female seeds) [7, 26]; and it is referred to as the ‘primary sex-ratio’ [3]. The deviation in ratio may be due to differential allocation of resources [13, 22, 26]. Males tend to require a lower amount of resource for growth and maintenance than females, and thus may have high survivability and longevity [3, 14, 27]. This may be due to greater allocation of resources required by the females to set fruits, and consequently female plants may exhibit greater mortality. The deviation in ratio leads to manifestation of ‘secondary sex-ratio’ which is driven by the life-history traits of species and demographic factors [3, 18, 19]. Recent studies have also highlighted the role of competition between X- and Y-carrying pollen grains, in context of population size and plant density, which may also influence the primary sex-ratio [28, 29].

*Hippophae rhamnoides* ssp. *turkestanica* (commonly known as Himalayan Seabuckthorn, family: Elaeagnaceae) is a perennial, woody, subdioecious, and anemophilous taxon. Our previous studies have established diploidy and apomixis in the species [30, 31]. The fruits of Seabuckthorn are rich in bioactive compounds, carbohydrates, vitamins, amino acids, essential fatty acids, and notable amount of oil which have anti-UV, skin regeneration and wound healing properties. The species also hold immense ecological values with its nitrogen fixation capacity, and clonal growth which helps in soil stabilization in the region [32, 33]. The natural populations of the species are present in vast geographical area of western Himalaya in varying habitats ranging from riverine area to dry regions. Our earlier studies had indicated an ongoing transition to dioecy via monoecy in the species due to the presence of polygamomonoecious (PGM) plants in natural populations [30, 31]. Though found in low frequency, PGM plants exhibit an important transitory state towards dioecy. Perusal of literature indicates that subdioecious taxa have been rarely investigated to understand the RA pattern and sexual dimorphism together. The present study is an attempt to fill this gap. More specifically, we investigated (i) the extent of sexual dimorphism in life-history traits of the species; (ii) resource allocation strategy of the sexes; (iii) sex-ratio in the natural populations occupying variable habitat conditions; and (iv) the effect of habitat on sex-ratios, sexual dimorphism, and RA in the species.

## Material and methods

### Study site

**Fig 1:**
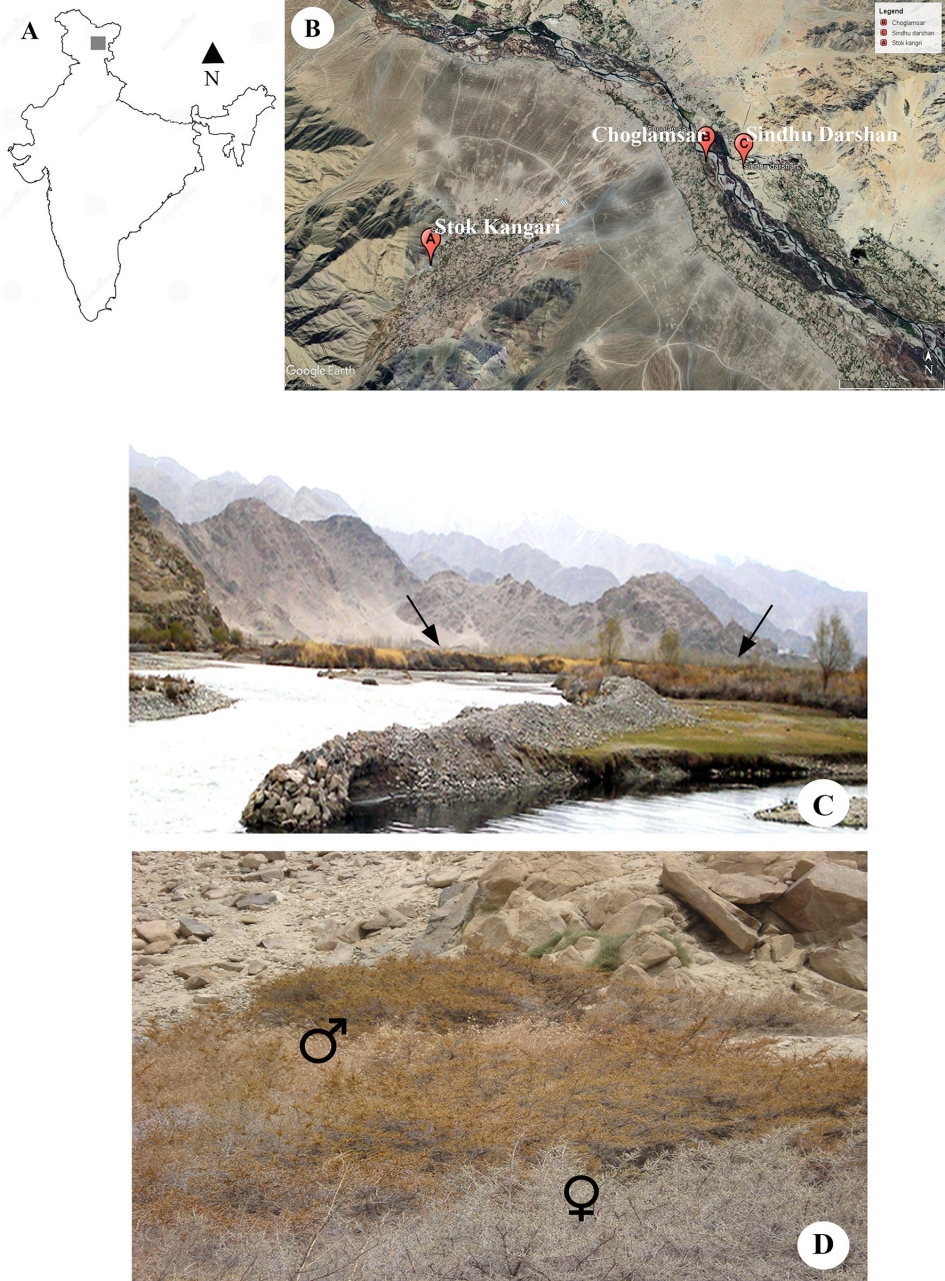
Location of the study site (A) and the populations (B). C-D: View of populations. C. The distant view of Choglamsar (CV) population (arrows) growing in riverine delta of river Indus. D. Part of population growing at hill slope of Sindhu Darshan (SD) as seen during flowering. Male and female patches are marked.

The study site is located in the Union Territory of Ladakh, western Himalaya, India. Three natural populations namely, Sindhu Darshan (SD), Choglamsar, (CV) and Stok-Kangari (SK) were selected for the study. The populations are present in contrasting habitat conditions and at different altitudes *viz*. CV occupies a riverine delta (n = ~950; N = 34°05.252’, E = 77°36.110’, 3240m above sea level), SD occurs on a dry slope (n = ~800; N = 34°05.269’, E = 77°36.714’, 3234m asl), and SK (n = ~180; N = 34°03.933’, E = 77°32.745’, 3545m asl) is located at dry area of highest altitude among all the three (Fig 1). Only 5 polygamomonoecious (PGM) plants were found in CV population, which were included in the study. The mean temperature at the study site ranged between -15°C to 10°C during the flowering (April-May) and 15°C to 30°C during fruiting (May-September) periods. The soil type ranges from sand-gravel at slopes while sand-silt at riverine plain [34]. Importantly, Seabuckthorn is the only species that grows at the selected sites, and is unlikely get influenced by competition-induced changes in growth attributes of the plants. The study was conducted for two consecutive seasons during 2021–2022.

### Sexual dimorphism

The terms sex and gender have been used interchangeably in literature. Here, we have used the term ‘sex’ to indicate the sexuality of plants and their functionality [35]. The dimorphism was recorded, both in the terms of vegetative as well as reproductive traits, for the male, female (n = 15 each), and PGM plants (n = 5). The vegetative characters included height of the plant, circumference of the main stem (first order), number of branches (second order stem), number of thorns, leaf-area, and leaf fresh-weight. The height and circumference of the main stem was recorded using a calibrated metre scale. The number of branches and thorns on the main stem were noted down. Leaf-area (n = 25 leaves, each sex) was calculated using ImageJ software [36]. Specific leaf-area was also calculated as the ratio of leaf-area to leaf dry-weight [21].

The reproductive traits studied included the average number of inflorescences in a plant, and were estimated as the multiplication product of the average number of inflorescences present per 10 cm of main stem and the total number of shoots (second order and thorns) on a plant. The number of flowers borne in an inflorescence (n = 150), dry-weight of the flowers (n = 225), and dry-weight of shoot were determined for all the sexes while data of fruits-set per infructescence (n = 225 from female, n = 30 from PGM), fresh- and dry-weight of fruit (n = 150 from female, n = 30 from PGM) was obtained.

### Resource allocation: Vegetative and reproductive

**Fig 2:**
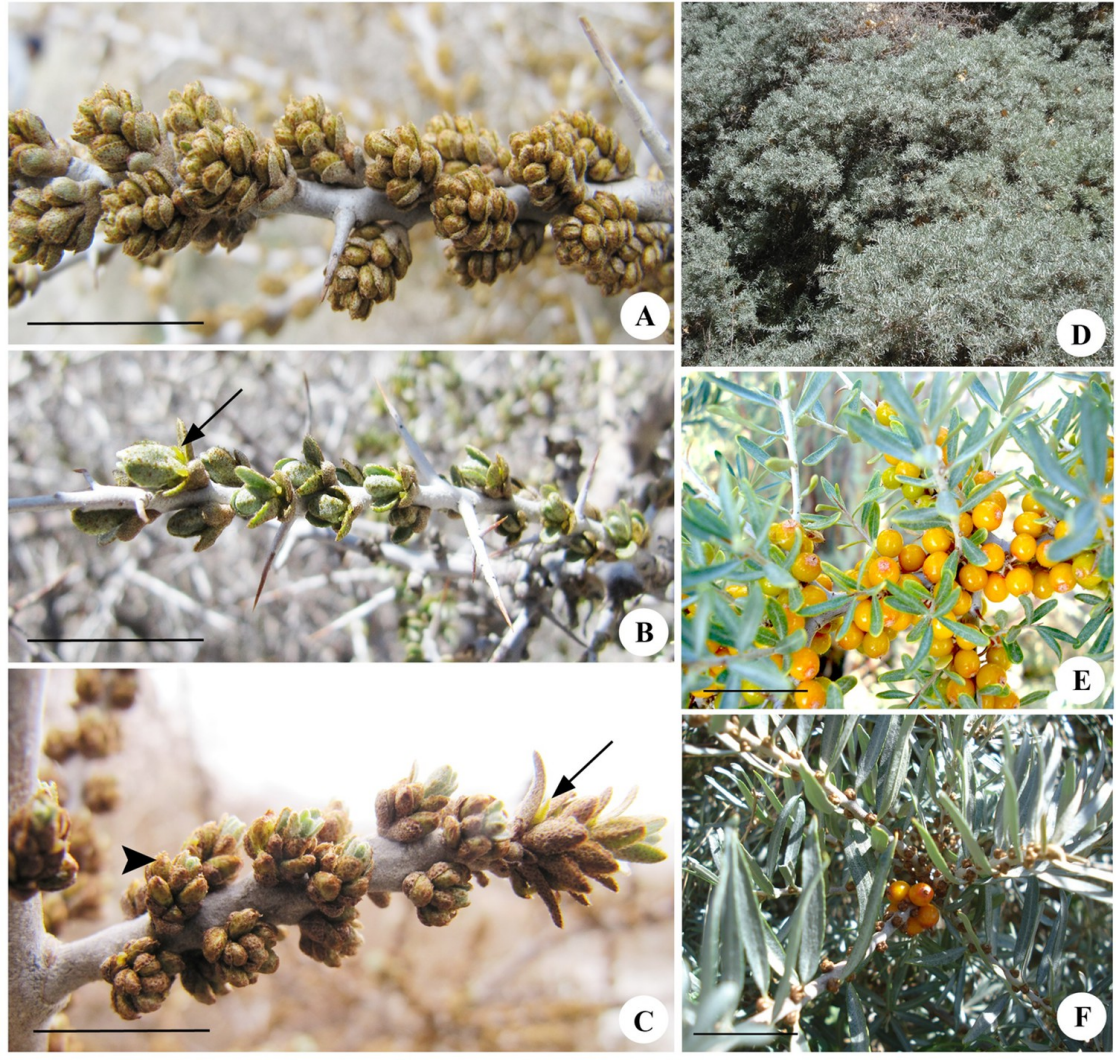
The male, female, and polygamomonoecious (PGM) plants of Seabuckthorn at the time of flowering (A-C) and fruiting (D-F). A. Male inflorescences. B. Female inflorescences. Arrow indicates the protruded stigma of a female flower. C. Inflorescences borne on a PGM plant with a female flower (arrow), and a male flower (arrowhead). D. Canopy of a male plant during August-September with leaves. E. Part of a canopy of female plant with numerous infructescences and fruits. F. PGM plant with fruits. Scale bars: A-C, E-F = 1 cm.

Resource allocation (RA) was estimated as the ratio of reproductive to vegetative dry-weight at the time of flowering (RA_fl_) as well as fruiting (RA_fr_) [37, 38]. To compute RA, from each of the three populations, we sampled ten male and ten female plants, along with five PGM plants from the CV population. During flowering, the plants were leafless, so, for estimating vegetative allocation during flowering, a shoot (~10 cm) with all its thorns and inflorescences was collected (Fig 2A–2C). To obtain dry-weight, the sampled tissues were oven-dried at 55°C. The reproductive and vegetative allocation was determined by weighing the dried inflorescences and shoots, separately.

During the fruiting season, the plants begin to exhibit vegetative growth, bear mature leaves and new shoots/thorns. As the plants bear foliage at the time of fruiting, they were sampled together. For the female and PGM plants, dry-weight of the fruits developed on the sampled shoots was taken for determining reproductive allocation (Fig 2D–2F).

The leaf samples of each sex were oven dried and analysed for the percent content of carbon, hydrogen, nitrogen, and sulphur using CHNS analyzer (Elementar Vario Micro Cube company, India). For CHNS analysis, dried leaf samples were ground into a fine powder (5–7 mg), mixed with 25–35 mg of tungsten hexa-oxide and then wrapped in a zinc foil before CHNS analysis [39].

### Sex-ratio

#### Primary sex-ratio

In order to determine the primary sex-ratio, the open-pollinated seeds were collected from the marked female plants (n = 10, each population), and about 15–20 seeds per plant were germinated in Soilrite® under laboratory conditions. A total of 100 seedings (10 from each plant, each population) were randomly collected and used to extract DNA by the protocol standardised earlier [40]. Seedling DNA was used as a template for PCR to check amplification with a previously developed male-specific SCAR marker (HRML). PCR reaction conditions were the same as used in an earlier investigation [31]. Amplification of Internal Transcribed Spacer (ITS1 and ITS4) region was used as a positive control to check the quality of DNA [41]. The seedlings DNA giving amplification with HRML marker were considered ‘male’ while those that did not as ‘female’ (*S1 Fig*).

In order to understand the difference between sexes in terms of height and weight of seedlings, seeds (n = 50) from females (n = 5) of each population were pooled, and germinated in Soilrite® mix. After 15 days of growth, the seedlings were carefully harvested along with their roots, and measured for their individual weight and height. Subsequently, the total genomic DNA was extracted followed by PCR amplification with HRML scar marker to assign sex to each seedling as detailed above.

#### Secondary sex-ratio

Seabuckthorn grows profusely by means of root suckers, thus forming aggregated ramets of each sex. In the present investigation, each flowering stem with ≥5 cm circumference was included to avoid over or under representation of one sex in a ramet [3]. The secondary sex-ratio i.e., the number of male plants per female was counted by employing the line-transect method during the flowering period of the species. Line transects of 10 m (n = 32, 32 and 20; at SD, CV and SK, respectively) were laid randomly to record data. A minimum distance between any two transects was about 15 m.

### Soil analysis

Soil samples were analysed to determine differences in nutrient availability among the male and female ramets’ rhizosphere in each habitat type. As the PGMs grow in close proximity of the other two sexes in CV population, separate sampling of soil was not done. The percent soil moisture content was recorded by inserting the moisture probe (Soil moisture meter, Lutron, USA) into the soil (5–20 at cm depth). Rhizosphere soil from the male and female plants was collected (n = 3–5) in plastic bags and then air-dried. For each sample, pH, electrical conductivity (EC), and salinity were recorded using a PCS tester (Eutech, India). For this, air-dried soil was sieved through 2 mm sieve and then soil suspension was prepared by adding 16 g of soil to 40 ml distilled water (1:2.5, *w*/*v*). The mixture was stirred for 15 min on a magnetic stirrer and the pH was measured. For EC and salinity, the suspension was filtered through Whatman no.1 filter paper, and the filtrate was used for measuring. For CHNS analysis, 10 g of air-dried soil sample was sieved through 2 mm sieve and 25–35 mg of the sieved soil was used.

### Statistical analysis

To test the variability between the seasons, populations, and the investigated traits, we applied multivariate analysis of variance (General Linear Model) by considering sexes as fixed factor and the seasons, populations, and traits as random factors. As the data did not differ by seasons, it was pooled population-wise and analysed to assess the sex-based differences between the populations. For computing the incidence of sexual dimorphism, the sexes were also analysed by combining the data from all the populations for each of the trait. The sexual dimorphism index (SDI) was calculated as log (*M*:*F*), where *M* and *F* refers to mean of each trait in the males and females, respectively [25, 42]. The significance of the SDI was calculated using the chi-square test. All statistical analyses were performed using SPSS software version 21 (IBM, 2012). The mean values are presented as mean ± standard deviation. Pearson Correlation was calculated for height and weight of seedlings with the sex, as deciphered by SCAR marker. The chi-square test was also done for the seedling height and weight, for both the sexes as well as the secondary sex-ratio. Principal component analysis (PCA) was performed to assess co-variability in traits with sexes, between populations & sexes, traits only, sexes and ecological factors. PCA was carried out using R software and the graphs were plotted using JJ-plot (R Core Team, 2023).

## Results

### Sexual dimorphism

**Fig 3:**
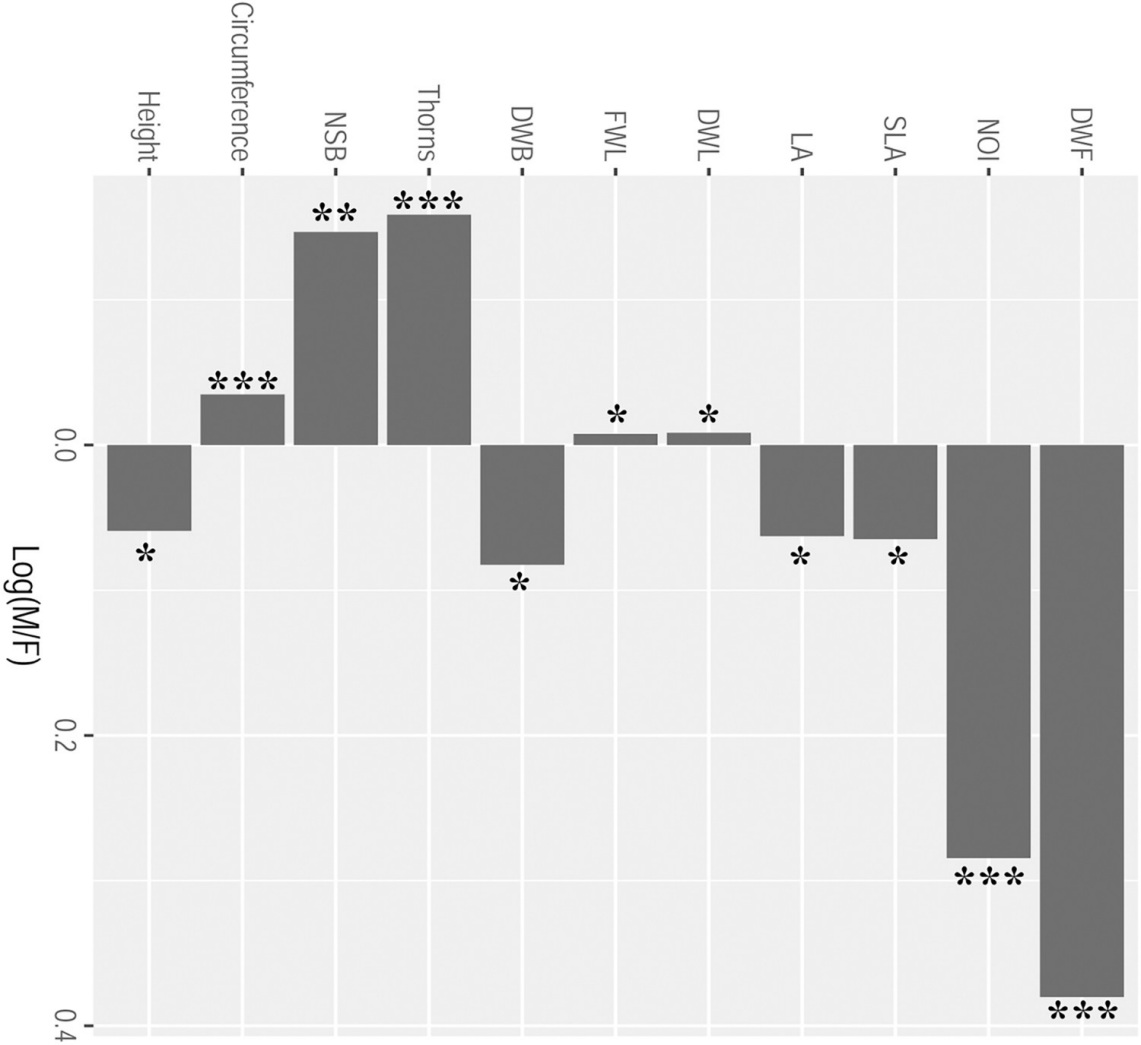
Sexual dimorphism and its indices for vegetative and reproductive traits in *Hippophae rhamnoides*. The sexual dimorphism index for each trait is variable from 0 and significant between male and female plants. Asterisks indicate the significance (*P < 0.05; **P < 0.01; and ***P < 0.001). Vegetative traits: Height, Circumference, NSB: number of sub-branches, Thorns, DWB: Dry-weight branch, FWL: Fresh -weight leaf, DWL: Dry-weight leaf, LA: Leaf-area, SLA: Specific leaf-area; Reproductive Traits: NOI: number of Inflorescence, DWF: Dry-weight flower.

The sexual dimorphism index for each trait was significantly variable from zero, and thus, significantly different between the male and the female plants (Fig 3). The vegetative traits *viz*. circumference of main stem (first order), number of branches (second order), thorns (third order) and fresh-weight of the leaves, were biased towards females while the traits *viz*. height, leaf-area, dry-weight leaf, dry-weight stem, and specific leaf-area were male-biased (Fig 3). The reproductive traits also showed significant dimorphism between male and female plants. Male plants produced two-fold inflorescences than the females, the mean dry-weight of male flowers was also higher than female flowers. Fruits-related traits like average fruit-set per infructescence, and fruit-weight were female-biased. Percentage total C and N content in the leaves did not vary significantly between the sexes as well as among the populations. The mean C content in the male and female leaves was 46.06±0.95 and 46.29±0.96, while mean N content was 10.21±0.16 and 10.08±0.17, respectively.

**Fig 4:**
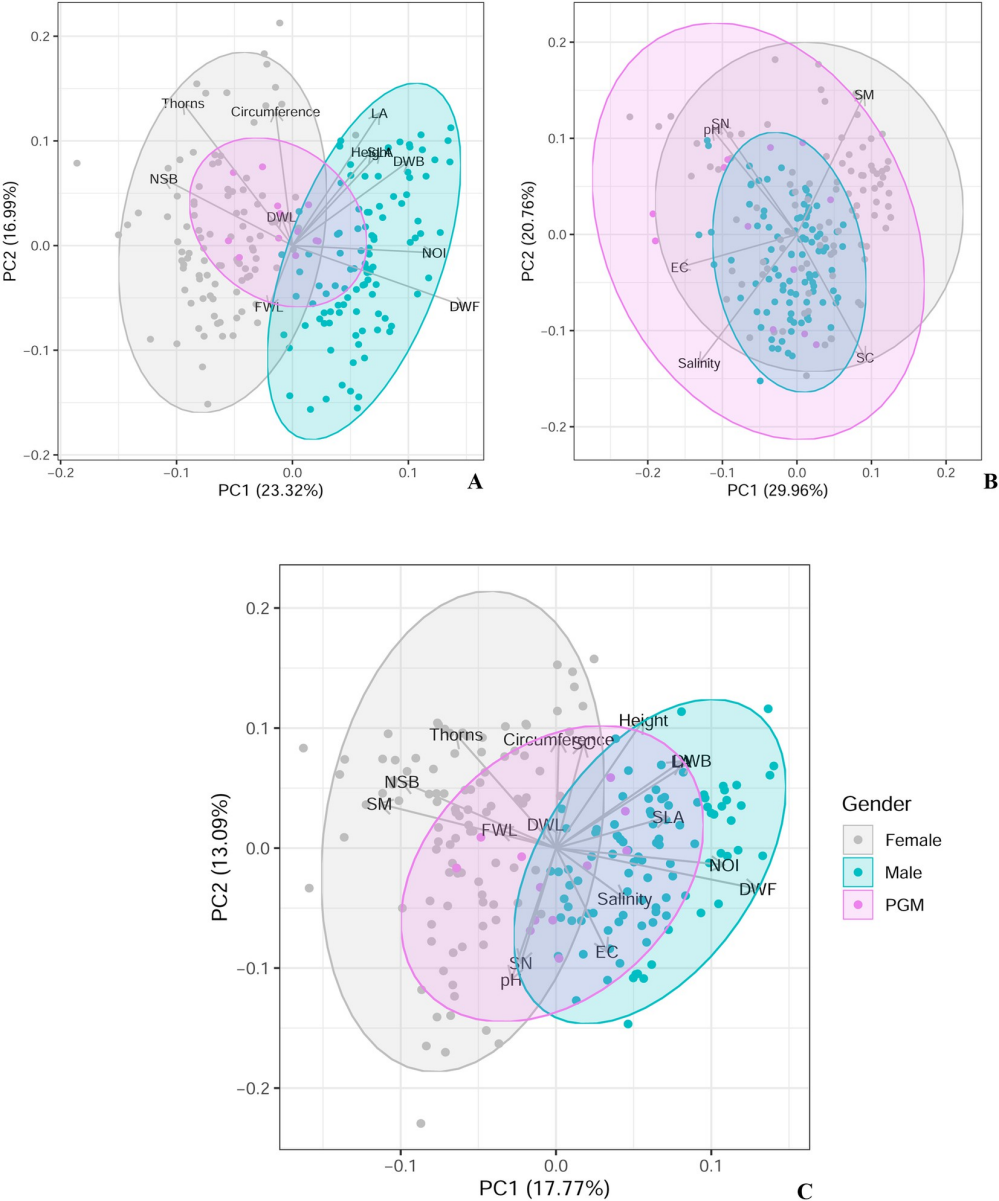
Principal component analysis for the traits of male, female and polygamomonoecious (PGM) plants along with the investigated abiotic factors. A. Traits and sexes. The results are same as calculated by Sexual Dimorphism Index. B. Edaphic factors and sexes. The plot clearly demonstrates that female rhizosphere soil possesses more moisture content. C. Traits, edaphic factors and sexes. The plots clearly depict the incidence of sexual dimorphism in *H*. *rhamnoides* and significantly biased traits to each sex (see vectors). Notably, the female sex is significantly co-variable with moisture content of soil. (NSB: number of sub-branches, DWB: Dry-weight branch, FWL: Fresh-weight leaf, DWL: Dry-weight leaf, LA: Leaf-area, SLA: Specific leaf-area; Reproductive Traits: NOI: number of Inflorescence, DWF: Dry-weight flower. SM: Soil moisture, EC: Electrical conductivity, SC: Soil Carbon, SN: Soil Nitrogen). *For separate PCA plots between populations, traits and sexes also see S2 Fig*.

**Fig 5:**
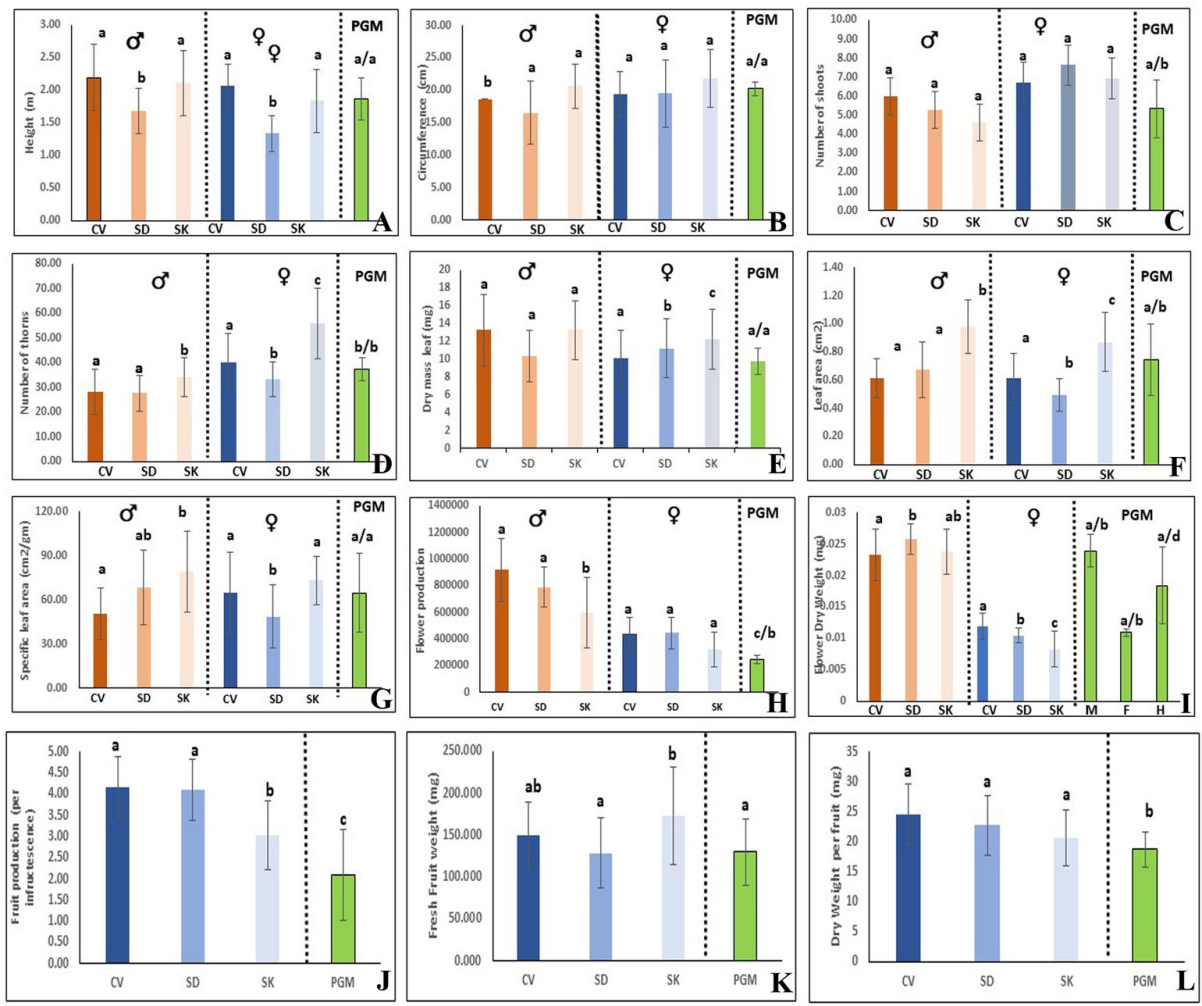
Bar graphs showing comparison of vegetative and reproductive traits studied in different populations and between sexes. A. Height, B. Circumference, C. Number of sub-branches, D. Number of thorns, E. Dry-weight leaf, F. Leaf area, G. Specific leaf area, H. Flower production, I. Dry-weight flower, J. Fruit production, K. Fresh weight fruit, and L. Dry-weight fruit. The letters in lower case over the bars, indicate the significant differences in trait between sexes (male, female and polygamomonoecious [PGM] separately) and between populations as obtained after post-hoc Tukey’s test.

The PCA analysis also showed that there is pronounced sexual dimorphism in vegetative and reproductive traits between male and female plants in the natural populations of the species (Fig 4). The vegetative traits of PGM plants either matches to males or to females and thus, were of intermediate to both the sexes (Figs 4 and 5). The reproductive traits, however, varied from those in male and female plants. In comparison to male and female plants, there was <50% flower production in the PGM plants. Fruit-set in PGM plants was also significantly lower than that in the female plants. The mean dry-weight of the fruits borne on the PGMs was also lower than fruits borne on the female plants (Figs 4C and 5).

#### Sexual dimorphism and effect of population

There is a limited extent of variability between populations for the traits investigated among the males and females (Fig 5, *S2A and S2B Fig*). For instance, male and female plants at SD populations were shorter in height than at CV and SK. The female plants at SK population had greater number of thorns, maximum leaf-size as well as the specific leaf-area (Fig 5). Among the three populations, mean dry-weight of the female flowers was highest in CV population. Although the populations did not differ in flower production among the females, fruit-set/infructescence production at SK was lower than that at CV and SD. Fresh-weight of fruits was highest at SK but there was no significant difference in dry weight per fruit among the three populations (Fig 5).

### Resource allocation: Vegetative and reproductive

Resource allocation (RA) during the flowering and fruiting periods varied significantly among the populations as well as between the male and female plants. As the populations did not differ by seasons for both the reproductive events (flowering and fruiting), the data was pooled (Table 1). During flowering, reproductive allocation (RA_fl_) was higher in males than females, owing to enormous flower production with more dry-weight. The male plants showed about two-fold resource allocation (dry-weight) in shoots (vegetative parts) at CV and SD population while it was about three-fold at SK population. The vegetative allocation was multi-fold in female plants than reproductive allocation at flowering. The total dry-weight of female flowers was least at SK population while it was highest at CV (Table 1). Overall, the male plants allocated greater amount of resources to vegetative parts too along with the reproduction as compared to female plants. PGM plants also exhibited higher resource allocation (two-fold) for shoots during flowering like to male plants.

**Table 1:** Summary of the reproductive dry-weight and vegetative dry-weight (g) of male and female plants in each population with results of multivariate analysis. In each sex, resource allocation (vegetative and reproductive) at flowering (RA_fl_), and fruiting (RA_fr_) can be calculated as the ratio of reproductive dry-weight to vegetative dry-weight.

Reproductive event	Dry-Weight	Population	Sex			Sex	Population	Population x Sex
			Male	Female	PGM			
Flowering	Inflorescence	CV	2.73±0.52	0.35±0.10	1.93±1.33			
		SD	2.15±0.55	0.26±0.07	-	*F* = 927.52, *P* = 0.000	F = 15.12, P = 0.000	F = 11.43, P = 0.000
		SK	1.91±0.84	0.15±0.06	-			
	Shoot	CV	5.84±1.45	5.04±1.56	5.25±1.02			
		SD	5.42±1.10	4.46±0.86	-	F = 30.64, P = 0.000	F = 4.66, P = 0.010	F = 0.748, P = 0.475
		SK	6.31±1.35	4.96±1.26	-			
Fruiting	Shoot+Leaves	CV	9.91±2.13	9.72±1.03	9.00±1.65			
		SD	10.79±2.44	10.05±0.76	-	F = 9.39, P = 0.002	F = 13.36, P = 0.000	F = 1.98, P = 0.14
		SK	12.06±1.45	10.68±1.98	-			
	Fruits	CV	-	7.80±0.86	0.32±0.03			
		SD	-	8.27±0.83	-	F = 6978, P = 0.000	F = 427.45, P = 0.000	F = 427.45, P = 0.000
		SK	-	3.16±0.43	-			

Reproductive allocation at the time of fruiting (RA_fr_) was significantly higher in the females than males (Table 1). The mean-weight of fruiting shoots in the females was notably higher than males. It was apparent that females allocated almost equal to reproductive and vegetative parts at CV and SD (RA_fr_ = 0.802 and 0.822 at CV and SD, respectively); this trend was lacking at SK population. Among the three populations, females at SK population exhibited the least number of fruits per shoot, and thus amounting to minimum RA_fr_ (about 1/3 of RA_fl_). However, cumulative vegetative allocation (dry-weight of leaves + shoot) in the females did not vary among the populations at the time of fruiting. Intriguingly, the mean dry-weight of male branch was maximum at SK population during fruiting as well. In PGM plants, the RA_fr_ did not differ either from the male or female plants. However, the allocation to fruit formation was lower than that in the females.

### Sex-ratio

#### Primary sex-ratio

Out of the 100 seedlings tested in each population, 54 conformed to male-specific (SCAR marker HRML) amplification at SD, 49 at CV and 56 at SK populations (*S1 Fig*). The chi-square test did not show significant differences (χ2 = 1.0438. p = 0.593405, p < 0.05) between male-specific amplification and no amplification (i.e., female). Thus, the primary sex-ratio was 1:1 (*M*:*F*). Height and weight of seedlings showed a significant positive correlation for males (Fig 4) i.e., male seedlings grows faster than female seedlings (*S3 Fig*). The chi-square test also showed a significant relationship between seedling sex (male) and height (χ2 = 14.942, *p* = 0.03675) and weight (χ2 = 50, *p* = 0.009) when tested separately.

#### Secondary sex-ratio

The populations differed in proportion of male and female plants (χ2 value = 20.4311, df = 2, *p* = 0.000037). Maximum number of male plants were at SD population while maximum females were present at CV. The CV population was female-biased in secondary ratio (0.435:1). In contrast, both SD (1.565:1.00) and SK (1.602:1.00) were male-biased.

### Soil analysis

PCA analysis of sex, their vegetative and reproductive traits (sexual dimorphism), and the edaphic factors clearly highlighted that these variables did not vary either by populations or the sexes, except soil-moisture content. The soil moisture content among the populations varied significantly; it was 0 at SK and maximum in CV (24%). In all the three populations, male plants proliferated under xeric conditions where the soil moisture content ranged from 0–5% while females preferred moist conditions. A significant positive correlation was observed between females and soil moisture content (Fig 4B and 4C, *S2C and S2D Fig*).

The total percent C and N content, pH, electrical conductivity (EC) and salinity also did not differ significantly among the sexes (*S2 and S4 Figs*). During flowering, CV population had lower total C content (male = 1.26±0.5, female = 1.2±0.4) than that at SD (male 4.12±2.8, female = 3.6±2.1 and SK (male = 6.7±3.5, female = 5.3±2.6). The total soil C content declined significantly in all the populations (and ranged between 3.323–4.478) at fruiting (September) than during the flowering season (April). A reverse trend was observed for the rhizospheric N-content (*S4C and S4D Fig*). During flowering, soil samples of male and female at CV population showed maximum total N content (male = 0.393±0.090, female = 0.323±0.250) while SD showed the least (male = 0.086±0.018, female = 0.126±0.162). The mean soil pH was 9.07 in samples from CV while at SD and SK, pH values were 7.66 and 7.09, respectively. There was no seasonal variation (flowering and fruiting) in soil pH among the populations (*S4E and S4F Fig*). The mean values of electrical conductivity (EC) and salinity in the soil declined during fruiting season (*S4G–S4J Fig*, *respectively*).

## Discussion

The present study highlights new insights into the extent of sexual dimorphism, resource allocation pattern among the sexes, and sex biasness under different habitat conditions of *Hippophae rhamnoides* in its natural distribution range. Similar earlier investigations on wind-pollinated taxa have largely included annuals such as gynodioecious *Mercurialis*, *Rumex*, and monoecious-dioecious *Sagittaria latifolia*, in which experimental manipulations are possible. Our study is among the limited studies on perennial and woody systems, and for the first time on a subdioecious system with polygamomonoecious (PGM) plants.

### Sexual dimorphism in Seabuckthorn

Sexual dimorphism in the life-history traits of dioecious taxa is known to maximise sexual functions [16–18]. Thus, the extent of sex-specific trait biasness may vary among the different plant species. In Seabuckthorn, the set of male-biased vegetative traits include height, leaf-area, specific leaf-area, leaf dry-weight, while circumference (main stem), number of branches and thorns corresponded with females. The reproductive traits like inflorescence and flower production linked significantly with the males. Such a bias in traits suggests a pronounced sexual dimorphism in the species, as supported by both Principal component analysis (PCA) analysis and the sexual dimorphism index. Being an anemophilous taxon [30], the larger size of males maximizes their reproductive output by enhanced pollen dispersal (or male sexual size dimorphism) [19, 38, 43]. Contrastingly, female plants of Seabuckthorn are shorter in height, has more circumference, greater number of shoots, as well as thorns on the branches. Such investment in vegetative growth seems necessary to support the mass of enormous fruits on a plant [44].

Sexual dimorphism in vegetative and reproductive traits is also associated with varying strategies of male and female functions in response to the environment and helps a species to adapt in varying environmental conditions (e.g., *Vallisneria* sp., *Mercurialis annua*, *Salix sp*. [44–46]. In the present study, out of the three natural populations investigated, overall, only the SK population (male-biased population) exhibited a limited extent of variability than the other two populations. At SK, females had lowest reproductive output (lowest fruit-set), and higher proportion of thorns than CV and SD. The magnitude of differences in the other traits (except fruit set and thorns) of the species did not show significant difference among the populations, thereby ruling out the incidence of phenotypic plasticity [43, 46–48]. The PCA analysis indicates that sex-based differences between male and female traits are, overlapped (i.e., Height, Circumference, FWL, DWL, and SLA) with PGM plants (Fig 4). Previous studies on *Hippophae rhamnoides* ssp. *sinensis* from Balang mountain in China have shown the effect of altitude and water stress on various leaf traits and physiology. Their study indicated sex-based differences (female-biased) in specific leaf-area, N content, height, and photosynthesis rate depending on altitude [49, 50]. However, we did not find any habitat-influenced bias in such traits of Seabuckthorn. These findings reiterate our previous conclusions of having limited differences between the genomes of sexes in the species, and of the ongoing evolution of dioecy in Seabuckthorn [51]. Thus, it is likely that sexual dimorphism in the species has recently evolved or ongoing in the species at the site.

### Resource allocation pattern

Dioecious plants may exhibit differences in resource allocation strategy at different stages of growth [10]. The timing of investment of resources in the two sexes is dynamic and may differ for vegetative growth and sexual reproduction [3]. This dynamism in Seabuckthorn can be understood in terms of resources allocation pattern to the vegetative and reproductive traits, both at the time of flowering and fruiting.

During flowering, male plants of Seabuckthorn seem to invest more in the reproductive structures (inflorescences and flowers), as indicated by almost two-fold production of inflorescences, and greater number of flowers than those in the females. However, there seems to be almost equal investment to the vegetative traits. On the other hand, during fruiting, the females allocated more for reproductive traits in the form of fruit formation. On the contrary, the investment is equal to the vegetative traits like photosynthetic tissue at this time, as indicated by similar specific leaf-area as well as C and N content. Interestingly, during fruiting, when females are expected to invest more resources towards fruit formation; females exhibited almost equal resource for vegetative function by producing more branches. These results are in agreement with other findings that establish functional role of sexes in terms of optimization of reproductive performance [10, 52, 53]. Thus, in Seabuckthorn there is sexual dimorphism in vegetative traits despite the absence of a bias in resource allocation. This is a deviation from other investigation, in which differential resource allocation is seen to cause sexual dimorphism [10, 21, 46].

The differential resource allocation pattern to each sex may also be governed by soil nutrient availability and its utilization. The females rely heavily on carbon and water for fruit/seed production while males invest more nitrogen for pollen production [3, 54]. In Seabuckthorn soil C and N did not vary among the sexes (both during flowering and fruiting), which highlights the absence of nutrient-based trade-off among the sexes for vegetative and reproductive functions [46, 55]. The overall decline in total C and N content in soil during fruiting is apparent as vegetative growth phase, fresh leaf-flush and fruiting occurs simultaneously in the species. This resource allocation pattern highlights that how the two sexes optimize their respective fitness under extreme environment.

### Subdioecy and resource allocation strategy

*Hippophae rhamnoides* ssp. *turkestanica* is a subdioecious species. Although the natural populations of Seabuckthorn harbour very few PGM plants, they represent the transitory phenotype in the evolutionary pathway [31]. Our earlier findings demonstrated that PGM plants allocate significantly lesser to reproductive function (pollen output, pollen viability and fertility) than males, and in comparison to the females, fruit-set is also low among the PGM plants. In the present study, assessment of vegetative traits of PGM plants with respect to males and females indicated that there is no significant difference among them. Thus, the PGM plants do not exhibit variability in vegetative characters among sexes but variability for reproductive traits do exist i.e., male vs. female function of PGM plants. It is established that among the dioecious taxa, resource allocation pattern during different phases of growth helps in elucidating the magnitude and possible direction of sex-specific selection [18, 25, 38, 56]. In PGM plants of Seabuckthorn, such a condition is expected where such balance in investment or equal resource allocation to both male and female function is necessary to meet the assumption of monoecy pathway [20, 31].

### Sex-ratio

Among the dioecious taxa, underlying sex-determination mechanism may also contribute to sexual dimorphism [7, 57] while resource allocation strategy, pollen competition etc. may modulate the sex-ratio in the progeny. In Seabuckthorn, sex-determination mechanism is sex chromosome based [58], and the primary sex-ratio of 1:1 is expected as also observed among the seeds/progeny.

During establishment of seeds in natural conditions, various factors like the competitive ability of sexes, mortality, their spatial segregation/preference to area depending on availability of resources, abiotic factors (soil, light etc.), and age of reproductive maturity may influence the secondary sex-ratio in natural populations [3, 13, 26]. The nutrient rich soils favour females to meet higher investment during reproduction [12, 59]. In Seabuckthorn, the three populations (CV, SK and SD) showed variable secondary sex-ratio, which could have been due to a variety of reasons [4, 9, 60, 61 and *references therein*]. Our results indicate that soil nutrient availability did not vary between sexes and there was no mortality of male and female plants/seedlings in natural populations. Among the edaphic factors studied, only moisture content showed significant relation with female sex. This was one of the defining features of the habitat that significantly corresponded with females (at CV), it may be inferred that it might be modulating the secondary sex-ratio in the species. The rhizosphere around the females also had more moisture than that of male ramets. This finding is in agreement with several studies where male-biased sex-ratio were reported under xeric and resource-limited environment condition, as in *Aralia nudicaulis*, *Pistacia chinensis*, and *Spinacia* sp., largely owing to their physiology [62–65]. Nonetheless, in a previous study on Chinese population of *H*. *rhamnoides* the authors have suggested that 2800 m altitude represents the range for optimal growth of *H*. *rhamnoides*; at higher altitudes water stress induces negative impact on females through poor growth and greater mortality [51]. Thus, it is likely that the biased sex-ratio may be associated with changing altitude, with SK at the higher elevation (≥300 m), which also conforms with findings in *Rumex nivalis* [28].

## Conclusion

Our study demonstrates that resource allocation pattern may vary by sexes in Seabuckthorn. Here, it was more pronounced towards the reproductive traits, which is in accordance with (i) the enhanced male fitness manifested through greater pollen production, as expected in many wind-pollinated dioecious taxa at the time of flowering, and (ii) greater allocation for fruit formation in the females at the time of fruiting. Although there is insignificant difference in biomass allocation among the sexes, there seems to be a trade-off in plant architecture of the two sexes for greater reproductive fitness. The vegetative trait such as greater height of the male plants would likely promote efficient pollen dispersal while shorter height and greater number of branches among the females would favour increased fruit-set. Also, moisture seems to regulate the dynamics of nutrient acquisition in favour of females while the males are adapted to colonize and may better survive under xeric conditions. Such suites of traits enhance the reproductive success of species inhabiting and an open and extreme environmental condition. In evolutionary context, the intermediate sexual phenotype (PGM) exhibits overlapping vegetative as well as reproductive traits which reiterates the monoecious pathway for the evolution of dioecy in the species. Seabuckthorn has a wide distribution range and how the species behaves in a geographic mosaic needs validation. Also, further investigations are needed to understand sexual dimorphism and resource acquisition strategies in relation to clonality, and underlying sex-determination mechanism in the species.

## Supporting information

S1 Fig Representative agarose gel profiles of SCAR marker amplification with seedlings DNA of the three populations. The seedling DNA with amplification (product size:~329bp) was counted as male while without amplification was marked as female in study.
(TIF)

S2 Fig A-B: PCA for populations and traits of sexes A. Male B. Female; C-D: PCA for populations and edaphic factors C. Male D. Female.
(JPG)

S3 Fig Pearson correlation plot of male and female seedlings with their height and weight. A positive correlation could be observed for male sex with height and weight.
(JPG)

S4 Fig Box plots depicting comparison of soil factors between male (white) female (black) along with the seasons flowering and fruiting.
(JPG)
