# Supplementary figures and images for: Variable resource allocation pattern, biased sex-ratio, and extent of sexual dimorphism in subdioecious Hippophae rhamnoides
Source: PLoS One. 2024 Apr 18;19(4):e0302211. doi: 10.1371/journal.pone.0302211 (PMC11025892; doi:10.1371/journal.pone.0302211)

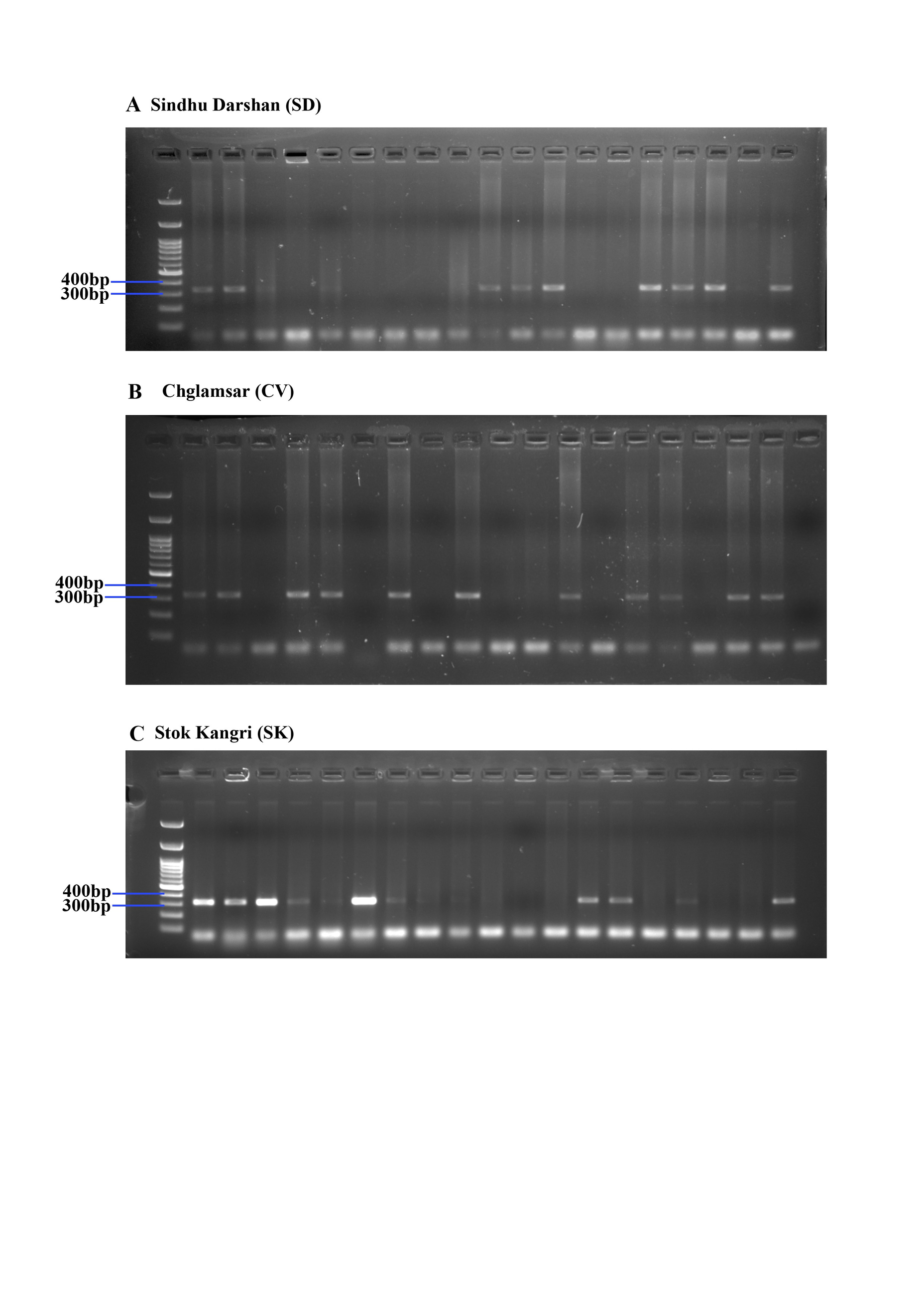

Supplement: S1 Fig — The seedling DNA with amplification (product size:~329bp) was counted as male while without amplification was marked as female in study. (TIF) [file pone.0302211.s001.tif]

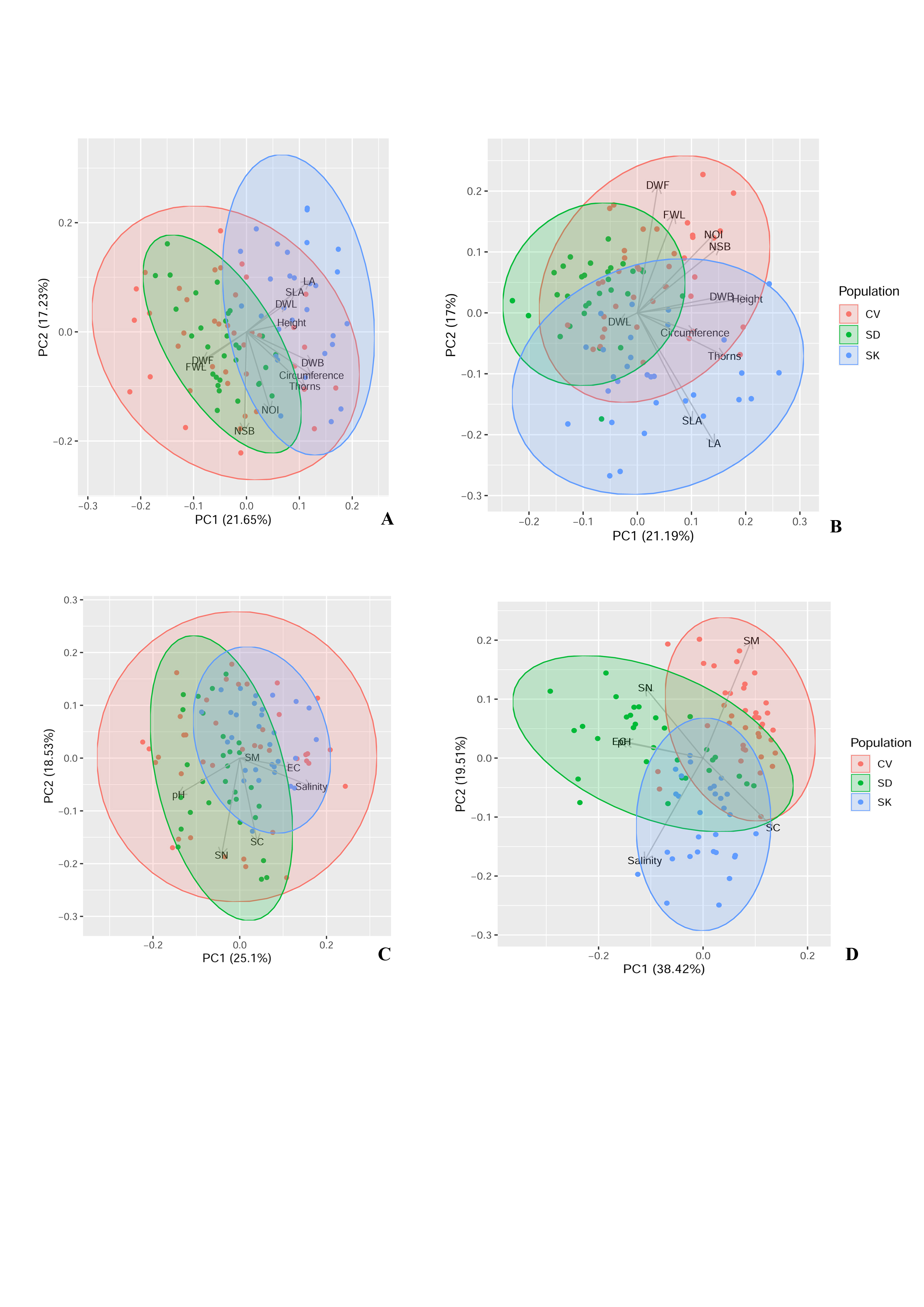

Supplement: S2 Fig — A-B: PCA for populations and traits of sexes A. Male B. Female; C-D: PCA for populations and edaphic factors C. Male D. Female. (JPG) [file pone.0302211.s002.jpg]

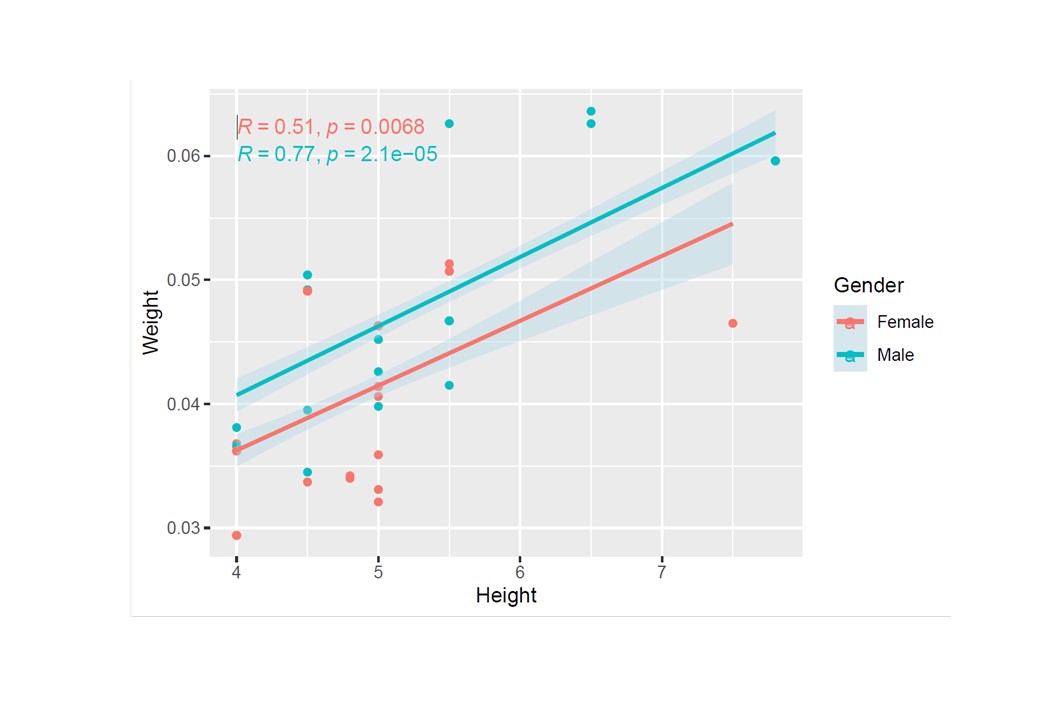

Supplement: S3 Fig — A positive correlation could be observed for male sex with height and weight. (JPG) [file pone.0302211.s003.jpg]

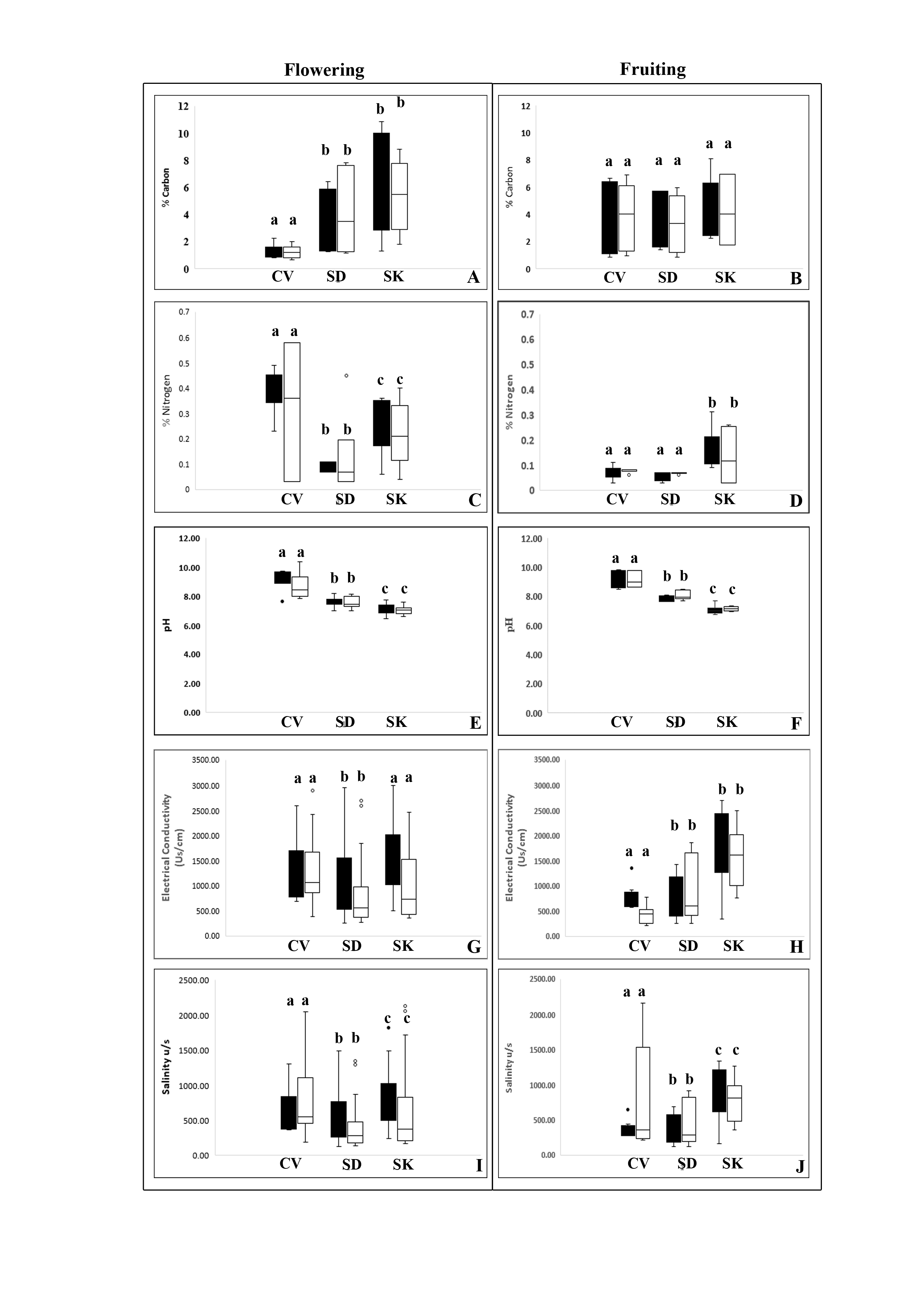

Supplement: S4 Fig — Box plots depicting comparison of soil factors between male (white) female (black) along with the seasons flowering and fruiting. (JPG) [file pone.0302211.s004.jpg]
